# tRNA queuosine modification is involved in biofilm formation and virulence in bacteria

**DOI:** 10.1093/nar/gkad667

**Published:** 2023-08-28

**Authors:** Jorge Díaz-Rullo, José Eduardo González-Pastor

**Affiliations:** Department of Molecular Evolution, Centro de Astrobiología (CAB), CSIC-INTA, Carretera de Ajalvir km 4, Torrejón de Ardoz 28850, Madrid, Spain; Department of Molecular Evolution, Centro de Astrobiología (CAB), CSIC-INTA, Carretera de Ajalvir km 4, Torrejón de Ardoz 28850, Madrid, Spain

## Abstract

tRNA modifications are crucial for fine-tuning of protein translation. Queuosine (Q) modification of tRNAs is thought to modulate the translation rate of NAU codons, but its physiological role remains elusive. Therefore, we hypothesize that Q-tRNAs control those physiological processes involving NAU codon-enriched genes (Q-genes). Here, we report a novel bioinformatic strategy to predict Q-genes, revealing a widespread enrichment in functions, especially those related to biofilm formation and virulence in bacteria, and particularly in human pathogens. Indeed, we experimentally verified that these processes were significantly affected by altering the degree of tRNA Q-modification in different model bacteria, representing the first report of a general mechanism controlling biofilm formation and virulence in Gram-positive and Gram-negative bacteria possibly through the coordination of the expression of functionally related genes. Furthermore, we propose that changes in Q availability in a microbiome would affect its functionality. Our findings open the door to the control of bacterial infections and biofilm formation by inhibition of tRNA Q-modification.

## Graphical Abstract

**Graphical Abstract:**
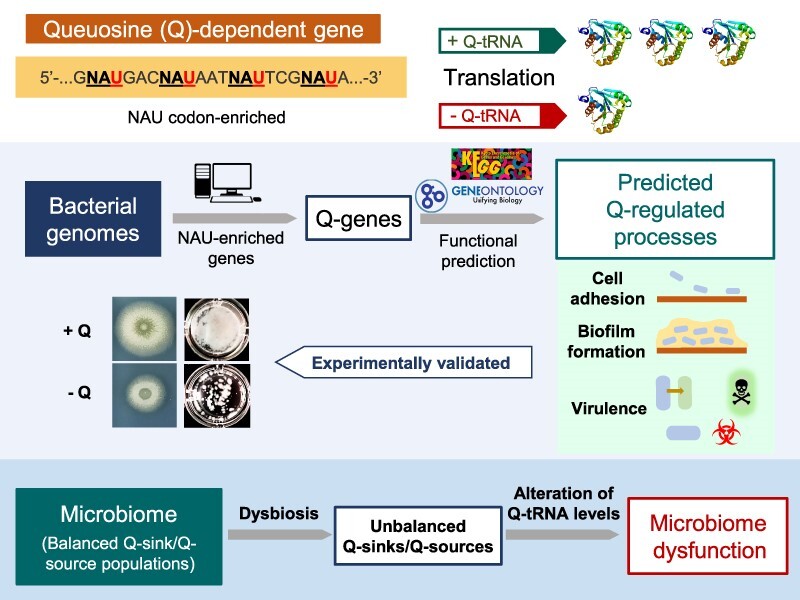

## INTRODUCTION

Translation of genetic information into proteins requires an efficient and accurate decoding of the mRNA by ribosomes and tRNAs. A wide variety of post-transcriptional modifications in tRNAs are critical for fine-tuning the translation process, representing an additional level of gene regulation (1). Despite their essential role in translation, the physiological function of several tRNA modifications is not very well understood. One such modification is queuosine (Q), a hypermodified nucleoside derived from guanine that is incorporated in the wobble anticodon position 34 of tRNAs containing the 5′-GUN-3′ anticodon sequence, those involved in decoding Asn, Asp, His and Tyr codons (AAC/U, GAC/U, CAC/U, UAC/U; NAC/U) (2).

**Figure 1.**
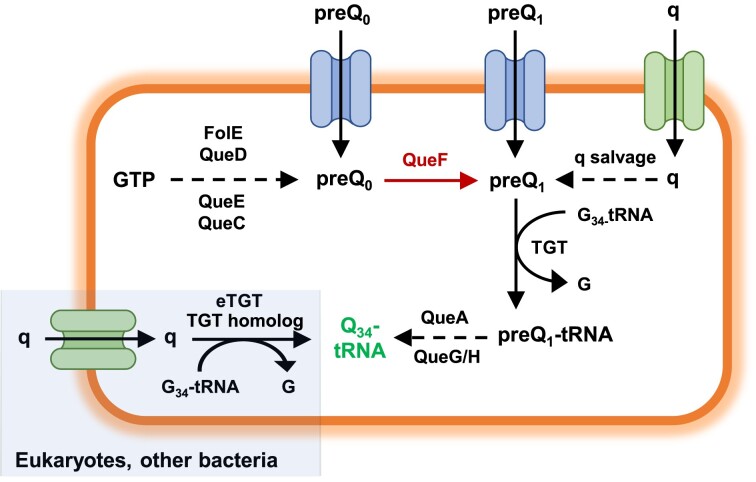
Q biosynthetic pathway in bacteria and eukaryotes. In bacteria, the Q biosynthesis pathway starts with five sequential modifications of GTP catalysed by the enzymes FolE, QueD, QueE, QueC and QueF to obtain preQ_0_ and preQ_1_ precursors. Then, preQ_1_ is incorporated into tRNA by tRNA guanine transglycosylase (TGT) and finally transformed into Q by QueA and QueG/QueH. Other bacteria cannot synthesize Q *de novo*: some species import preQ_0_ and preQ_1_ precursors through the YhhQ transporter, and others import q, which can be transformed into the preQ_1_ precursor or is directly incorporated by a TGT into tRNA and transformed into Q (q salvage). Eukaryotes cannot produce Q *de novo*, therefore they use q to produce Q-tRNA. In this work, the *queF* gene was deleted in *E. coli* and *B. subtilis* to obtain non-Q-producing strains (in red). G, guanine; eTGT, eukaryotic TGT.

Q is found in Bacteria and Eukarya, although its *de novo* biosynthesis only occurs in Bacteria. The Q biosynthesis pathway starts with five sequential modifications of GTP catalysed by the enzymes FolE, QueD, QueE, QueC and QueF to obtain the preQ_1_ precursor (3). This precursor is incorporated into tRNA by tRNA guanine transglycosylase (TGT) and finally transformed into Q by QueA and QueG/QueH (Figure 1) (4,5). Although many species can synthesize Q *de novo*, salvage of Q precursors also occurs. Some species use the YhhQ transporter for importing preQ_0_ and preQ_1_ precursors (5). Certain bacteria capture queuine (q), the Q nucleobase, and transform it into preQ_1_. Other bacteria and eukaryotes directly replace the guanine at position 34 of the tRNAs with q by using a TGT homologue or a eukaryotic TGT (eTGT), respectively (Figure 1). Bacteria that cannot produce Q *de novo* and eukaryotes need to salvage Q precursors through the microbiome and/or nutrient sources (6,7).

Since the discovery of tRNA Q-modification, multiple studies conducted in eukaryotes have revealed its involvement in very diverse processes including pupae maturation in *Drosophila melanogaster*, cell aggregation in *Dictyostelium discoideum*, and the antioxidant defence system, hypoxia, cancer and proliferation in mammals (8). However, in bacteria, only a few studies shed light on the physiological function of Q. Specifically, it has been reported that this modification regulates the virulence of the pathogen *Shigella flexneri* and the nodule cell infection efficiency of *Sinorhizobium meliloti* (9,10). Furthermore, our group showed that the overexpression in *Escherichia coli* of Q biosynthetic genes isolated from environmental microorganisms using functional metagenomics increased the resistance to several stressors such as heat shock, low acidic pH, UV radiation, perchlorate and arsenic (11,12).

The molecular mechanism underlying these widely diverse and spread phenotypes is not well understood. One explanation could be that Q has been shown to influence codon–anticodon interaction, which may affect the translation of certain genes (13). Previous studies performed *in silico* or in artificial eukaryotic systems demonstrated that G_34_-tRNAs harbouring the GUN anticodon show a strong preference for NAC codons over NAU codons, whereas Q_34_-tRNAs exhibit no bias for either cognate codon (14,15). Furthermore, Q-tRNAs have been reported to accelerate translational speed at NAU codons in mammals (16). Therefore, the presence of Q in tRNAs may prevent the translational codon bias shown by unmodified tRNAs and could increase the translational efficiency by enhancing translational speed at NAU codons. In this sense, Q modification of tRNA is known to be impaired in cancer cells, and *in silico* analysis revealed divergences in NAU codon usage between certain genes coding for housekeeping or oncodevelopmental proteins (15). In addition, in human cells cultured in the absence of q, an enrichment or depletion in NAU codons was observed in genes coding for down- or up-regulated proteins, respectively (16). Furthermore, Q-dependent translational regulation of a synthetic gene enriched for NAU codons *in vivo* in the eukaryote *Trypanosoma brucei* was recently reported (17). In summary, all these results suggest that availability of Q would particularly affect the translation of genes enriched in NAU codons, leading to a variation in the expression levels of the proteins they encode depending on the degree of Q-modification of tRNAs, at least in eukaryotes (15–17). In this respect, this general mechanism of regulation could be responsible for the wide variety of reported Q-related phenotypes, which may vary depending on the roles of the specific NAU codon-enriched genes in each organism.

In the present work, we first experimentally demonstrate that the Q-modification of tRNAs in bacteria affects the expression of NAU codon-enriched genes (Q-genes). Then, we have developed a bioinformatic analysis for the identification of Q-genes and for the prediction of the physiological effects of Q in a wide variety of bacteria, in which the role of Q is particularly unknown. We report that most bacterial species across all phyla, including Gram-negative and Gram-positive bacteria, harbour Q-genes, which are particularly involved in cell adhesion, biofilm formation and virulence. Indeed, we experimentally verified that these processes are greatly affected by Q-modification of tRNAs in the model bacteria *E. coli*, *Bacillus subtilis* and *Pseudomonas putida*. In addition, bioinformatic and experimental data highly suggest that Q enhances the virulence of most human pathogenic bacteria. Moreover, we propose a relationship between Q availability and the functionality of complex microbial communities, such as the gut microbiome.

## MATERIALS AND METHODS

### Bacterial strains, media and culture conditions

Bacterial strains used in this work were *E. coli* DH10B (Invitrogen), *E. coli* ST131 (kindly provided by Dr. Rosa del Campo, Hospital Ramón y Cajal, Madrid, Spain), *B. subtilis* PY79 laboratory strain (prototroph, derived from *B. subtilis* strain 168, from P. Youngman, University of Georgia, Athens, GA, USA), *B. subtilis* NCIB 3610 ‘undomesticated’ strain [from A. L. Sonenshein and the Bacillus Genetic Stock Center (BGSC), Ohio State University, Columbus, OH, USA] and *P. putida* KT2440 (kindly provided by Dr. Victor de Lorenzo, Centro Nacional de Biotecnología, Madrid, Spain). Other strains used in this work are described in Supplementary Table S1. Bacteria were routinely grown in Luria–Bertani (LB) medium. Minimal media used were M63 medium [100 mM KH_2_PO_4,_ 15 mM (NH_4_)_2_SO_4,_ 1.8 μM FeSO_4_] supplemented with 1 mM MgSO_4_, 0.2% (w/v) glycerol and 0.5% (w/v) casamino acids, and MSgg medium [5 mM potassium phosphate (pH 7), 100 mM MOPS (4-morpholinepropanesulphonic acid) (pH 7), 2 mM MgCl_2_, 700 μM CaCl_2_, 50 μM MnCl_2_, 50 μM FeCl_3_, 1 μM ZnCl_2_, 2 μM thiamine, 0.5% glycerol, 0.5% glutamate, 50 μg/ml tryptophan, 50 μg/ml phenylalanine, 50 μg/ml threonine] (18). When required, antibiotic final concentrations were 100 μg/ml ampicillin (AMP), 50 μg/ml kanamycin (KAN), 0.5 μg/ml erythromycin plus 2.5 μg/ml lincomycin (MLS), 100 μg/ml spectinomycin (SPT), 2 μg/ml tetracycline (TET), 2 μg/ml chloramphenicol (CHL) and 50 μg/ml streptomycin (STR). *E. coli* and *B. subtilis* strains were routinely grown at 37°C, and *P. putida* strains at 30°C. For solid cultures, the growth medium was supplemented with agar (15 g/l). Liquid cultures were shaken on an orbital platform operating at 200 rpm.

### Construction of a Δ*queF* mutant and strains overexpressing Q biosynthetic genes in *E. coli*

Q biosynthetic genes *queD*, *queE*, *queC*, *queF*, *tgt*, *queA* and *queG* from *E. coli* DH10B and *queF* from *B. subtilis* PY79 strain were cloned and overexpressed in *E. coli* DH10B, and *queF* from *E. coli* DH10B was also overexpressed in *E. coli* ST131. Genes were cloned into pBlueScript II SK (+) (pSKII+) using specific primers (Supplementary Table S2). The Q-deficient strain *E. coli* DH10B Δ*queF* was constructed by specific exchange of *queF* with an antibiotic resistance cassette by homologous recombination *in vivo*, using the Counter-Selection BAC Modification Kit and Red/ET recombination (Gene Bridges). Briefly, *E. coli* DH10B were transformed with the pRedET plasmid (Gene Bridges), which carries the λ phage γβα operon under the control of the arabinose-inducible pBAD promoter and confers TET resistance. A DNA cassette including a KAN resistance cassette and 65 bp flanking homology regions of *queF* was designed and synthetized (IDT; Coralville, IA, USA). This DNA cassette was introduced into *E. coli* DH10B pRedET cells by electroporation, and homologous recombination was induced by incubation in LB supplemented with 0.4% arabinose for 1 h at 37°C. Positive mutant clones were selected in LB-KAN plates, and correct insertion of the DNA cassette and deletion of *queF* was verified by polymerase chain reaction (PCR) and sequencing.

### Construction of a Δ*queF* mutant and complementation in *B. subtilis*

Deletion of the *queF* gene in *B. subtilis* strains NCIB 3610 and PY79 was achieved by the long-flanking homology PCR (LFH-PCR) strategy (19). This technique is based on the deletion of a target gene by inserting an antibiotic resistance cassette, in this case, MLS. The 1 kb flanking homology regions of *queF* were amplified by PCR, using primers with a 23 nt fragment that hybridize to the ends of the MLS resistance cassette (Supplementary Table S2). After purification, the PCR products were used as primers to amplify the MLS resistance cassette from the plasmid pDG646 (20). The PCR amplification program used was as follows: 1 cycle of 2 min at 94°C; 10 cycles of 30 s at 94°C, 30 s at 63°C and 6 min at 68°C; 20 cycles of 30 s at 94°C, 30 s at 63°C and 6 min plus 20 s per cycle at 68°C; and, finally, 1 cycle of 10 min at 68°C. The resulting DNA fragment is the Δ*queF::mls* deletion construct, which was introduced into *B. subtilis* by natural competence under the following conditions: a *B. subtilis* liquid culture grown on LB at 30°C overnight was diluted to achieve an OD_600_ = 0.08 in 10 ml of Modified Competence Medium (MCM), and was incubated at 37°C and 200 rpm. At the beginning of the stationary phase (OD_600_ = 1.5–2), 10 μg of deletion construct were added to 1 ml of the culture. After incubation at 37°C and 200 rpm for 2.5 h, cells were plated in the presence of MLS for selection of deletion mutants (21). The introduction of the Δ*queF::mls* mutation into the genome was confirmed by PCR analysis and sequencing.

To complement the deletion mutant, a copy of the *queF* gene was integrated at the *amyE* locus, coding for a non-essential α-amylase. First, the *queF* gene of the *B. subtilis* NCIB 3610 strain was amplified by PCR using flanking primers containing restriction sites at their 5′ ends (Supplementary Table S2). The PCR amplification product was purified from a 1% low-melting agarose gel using the QIAquick Gel Extraction kit (QIAGEN), digested with the appropriate restriction enzymes and cloned into pDR111, a vector for ectopic integration by a double recombination event at the *amyE* locus (originally obtained from David Rudner, Harvard University). *E. coli* DH10B cells were transformed with the ligation product, and the resulting recombinant clones were selected on LB-AMP agar plates and confirmed by restriction analysis and sequencing. Plasmid harbouring the *queF* gene was purified from *E. coli* DH10B transformants, linearized with SacI (New England Biolabs) to favour double recombination events, and introduced into *B. subtilis* NCIB 3610 Δ*queF::mls* by transformation as explained above (21). Transformants were selected in LB-SPT plates supplemented with 10 mg/ml starch. A positive iodine staining of transformants was indicative of the absence of α-amylase (AmyE) activity and thus of the insertion into the *amyE* locus by double recombination of the *queF* gene (20). The *amyE* locus of the selected transformant *amyE*::*queF* was amplified by PCR to verify that plasmid integration occurred correctly.

### Overexpression of the *queF* gene in the *P. putida* KT2440 strain

To overexpress QueF in the *P. putida* KT2440 strain, its corresponding gene was cloned into pSEVA2313 plasmid as described above for the *E. coli* DH10B strains, and the construct was then introduced in *P. putida* KT2440 by electroporation (22). Recombinant clones were selected on LB-KAN agar plates and confirmed by restriction analysis and sequencing.

### Codon-specific GFP reporter assay

Cognate NAC/U codons of the gene that codes for enhanced green fluorescent protein (EGFP) were all changed either to NAC (C-EGFP) or to NAU (U-EGFP) codons. These genes were chemically synthesized together with a strong constitutive promoter at the 5′ end to increase transcription (23) (IDT; Coralville, IA, USA). Both constructs were digested and ligated into pSKII+ or pACYC184 plasmids. pSKII+ ligation products were used to transform *E. coli* DH10B and DH10B Δ*queF* cells, and recombinant clones were selected on LB-AMP agar plates supplemented with 40 μg/ml 5-bromo-4-chloro-3-indolyl-β-d-galactopyranoside (X-Gal; Fisher Scientific) and 0.1 mM isopropyl-β-d-1-thiogalactopyranoside (IPTG; Roche), and confirmed by sequencing. pACYC184 ligation products were introduced into *E. coli* DH10B pSKII+/*queF* cells, and recombinant clones were selected on LB-AMP-TET-CHL agar plates and confirmed by sequencing.

For the fluorescent reporter assay, liquid cultures of *E. coli* DH10B and DH10B Δ*queF* cells harbouring pSKII+/GFP constructs, and *E. coli* DH10B pSKII+/*queF* cells with pACYC184/GFP constructs were grown until exponential phase at 37°C 200 rpm in LB-AMP or LB-AMP-TET-CHL, respectively. Aliquots of 1 ml of strain cultures were washed twice with M63 to remove Q precursors from the LB medium. Cells were diluted 1/100 in 5 ml of M63-AMP or M63-AMP-TET-CHL and grown overnight at 37°C 200 rpm. Cultures were adjusted to OD_600_ of 0.02 in M63-AMP or M63-AMP-TET-CHL and incubated at 37°C for 24 h. The DH10B Δ*queF* strain was grown in the absence or presence of 100 nM preQ_1_ (Sigma-Aldrich). After incubation, OD_600_ was measured and BugBuster® reactive (Novagen) was used to prepare protein extracts from 1 ml of cell cultures. A 200 μl aliquot of protein extracts was used to measure the fluorescence in a Qubit™ 3 Fluorometer (Invitrogen). The fluorescence/OD_600_ ratio was calculated, and values were further normalized against the clone of each strain harbouring the C-EGFP construct.

### LC ESI-MS/MS proteomics

*E. coli* DH10B and *E. coli* DH10B Δ*queF* pre-cultures were grown to reach exponential phase (OD_600_ = 1) in M63 liquid medium at 37°C 200 rpm. Three 20 ml cultures of each strain with grown pre-cultures diluted in M63 to an adjusted OD_600_ of 0.05 were incubated at 37°C 200 rpm for 6 h to reach stationary phase (OD_600_ = 3.5). Samples of 2 ml were centrifuged at 13 200 rpm for 2 min and supernatants were totally discard. Cell pellets were stored at –80°C for further proteomic processing.

Each cellular pellet was dissolved with lysis buffer containing 5% sodium dodecylsulphate (SDS; Sigma-Aldrich), 100 mM triethylammonium bicarbonate (Thermo Fisher Scientific) and a protease/phosphatase inhibitor cocktail (Thermo Fisher Scientific). Cell rupture and homogenization were achieved with the aid of a Potter homogenizer. Samples were reduced and alkylated by adding 5 mM Tris (2-carboxyethyl)phosphine and 10 mM chloroacetamide for 30 min at 60°C and homogenized by micro tip probe ultrasonication for 1 min on a UP50H ultrasonic lab homogenizer (Hielscher Ultrasonics). The homogenate was centrifuged at 16 000 *g* for 15 min at 4°C, and the supernatant containing the solubilized proteins was used for further analysis. Protein concentration was estimated by Pierce 660 nm protein assay (Thermo Fisher Scientific).

Protein digestion in the S-Trap filter (Protifi, Huntington, NY, USA) was performed following the manufacturer's procedure with slight modifications. Briefly, 50 μg of protein of each sample was diluted to 40 μl with 5% SDS. Afterwards, 12% phosphoric acid and then seven volumes of binding buffer (90% methanol, 100 mM TEAB) were added to the sample (final phosphoric acid concentration: 1.2%). After mixing, the protein solution was loaded to an S-Trap filter in two consecutive steps, separated by a 2 min centrifugation at 3000 *g*. Then the filter was washed three times with 150 μl of binding buffer. Finally, 1 μg of Pierce MS-grade trypsin (Thermo-Fisher Scientific) in 20 μl of a 100 mM TEAB solution was added to each sample in a 1:20 (w/w) ratio and spun through the S-Trap prior to digestion. Flowthrough was then reloaded to the top of the S-Trap column and allowed to digest in a wet chamber at 37°C overnight. To elute peptides, two stepwise buffers were applied (40 μl of 25 mM TEAB, and 40 μl of 80% acetonitrile and 0.2% formic acid in H_2_O), separated by a 2 min centrifugation at 3000 *g* in each case. Eluted peptides were pooled, and vacuum centrifuged to dryness.

The resulting peptides were subsequently labelled using the TMT-sixplex Isobaric Mass Tagging Kit (Thermo Scientific, Rockford, IL, USA) according to the manufacturer's instructions. After labelling, samples were pooled, evaporated to dryness and stored at –20°C until the liquid chromatograhy (LC)−mass spectrometry (MS) analysis. Three biological replicates of each condition were analysed.

bRP C18 fractionation of the TMT-labelled peptides was performed using a Stage-Tip with 12 punches of sulphonated divinylbenzene (CDS Empore™ SDB-RPS, Sigma-Aldrich). A step gradient of increasing acetonitrile concentrations (0–60% ACN) in a volatile high pH elution solution [10 mM ammonium formate (NH_4_HCO_2_), pH 10.0] was applied to the column to elute bound peptides into 10 different fractions collected by centrifugation. Finally, fractions were pooled into five fractions using the fraction mixing strategy *n* + 5 (i.e. fractions 1 + 6, 2 + 7, 3 + 8, 4 + 9 and 5–10). The peptide fractions were dried, desalted using a Stage-Tips C18 (3M) and stored at −20°C until the LC-MS analysis.

After fractionation, peptide concentration was carried out by Qubit™ Fluorometric Quantitation (Thermo Fisher Scientific). A 1 μg aliquot of each fraction was subjected to 1D-nano LC ESI-MS/MS (liquid chromatography electrospray ionization tandem mass spectrometric) analysis using an Ultimate 3000 nano high-performance liquid chromatography (HPLC) system (Thermo Fisher Scientific) coupled online to a Orbitrap Exploris 240 equipped with a FAIMS Pro ion source (Thermo Fisher Scientific). Peptides were eluted onto a 50  cm  × 75 μm Easy‐spray PepMap C18 analytical column at 45°C and were separated at a flow rate of 300 nl/min using a 90 min gradient ranging from 2% to 95% mobile phase B [mobile phase A, 0.1% formic acid (FA); mobile phase B, 80% ACN in 0.1% FA]. The loading solvent was 2% ACN in 0.1% FA and the injection volume was 5 μl.

Data acquisition was performed using a data-dependent top 20 method, in full scan positive mode, scanning 375–1200 *m/z*. Survey scans were acquired at a resolution of 60 000 at *m/z* 200, with a normalized automatic gain control (AGC) target (%) of 300 and a maximum injection time (IT) in AUTO. The top 20 most intense ions from each MS1 scan were selected and fragmented via higher-energy collisional dissociation (HCD). Resolution for HCD spectra was set to 45 000 at *m/z* 200, with an AGC target of 100 and a maximum ion IT in AUTO. Isolation of precursors was performed with a window of 0.7 *m/z*, exclusion duration (s) of 45 and the HCD collision energy was 30. Precursor ions with single, unassigned or six and higher charge states from fragmentation selection were excluded.

### Proteomics data analysis and sequence search

Raw instrument files were processed using Proteome Discoverer (PD) version 2.4 (Thermo Fisher Scientific). MS2 spectra were searched using four search engines (Mascot v2.7.0, MsAmanda v2.4.0, MsFragger v3.1.1 and Sequest HT) and a target/decoy database built from sequences in the *E. coli* (strain K12) proteome at Uniprot Knowledgebase (20210222). All searches were configured with dynamic modifications for TMT reagents (+229.163 Da) on lysine and N-termini of the peptide, pyrrolidone from Q (–17.027 Da) and oxidation of methionine residues (+15.9949 Da) and static modification as carbamidomethyl (+57.021 Da) on cysteine, monoisotopic masses and trypsin cleavage (maximum two missed cleavages). The peptide precursor mass tolerance was 10 ppm, and MS/MS tolerance was 0.02 Da. The false discovery rate (FDR) for proteins, peptides and peptide spectral matches (PSMs) peptides were kept at 1%. The quantification values for proteins were calculated using the abundance of total peptide for the identification of differentially expressed proteins (DEPs). In this case, the peptide group abundances were summed for each sample and the maximum sum for all files was determined. DEPs were extracted by performing two-sided, two-sample *t*-test followed by FDR correction (α = 0.05) (24). Proteins with an associated *P*-adjusted value < 0.1 were considered as DEPs (Supplementary Data S1). The Kazusa database was used to obtain codon data of all *E. coli* genes (25). The frequency of NAU codons of each gene was calculated dividing the number of NAU codons by the total number of codons. The average frequencies of NAU codons of genes encoding up- and down-regulated proteins were compared with the average frequency of NAU codons in the whole genome using one-tail *t*-test.

### Bioinformatic analysis of Q-genes

Codon data were retrieved from the coding sequence (CDS) genomic Reference Sequence (RefSeq) database at NCBI and the Kazusa ddatabase (25,26). CDSs were associated with their corresponding UniProt IDs using the conversion tool available at the UniProt website. Only CDSs with an associated UniProt ID were considered for calculations. If a UniProt ID was associated with several CDSs, only one CDS was employed in the analysis. The frequency of NAU codons was calculated for each gene and microorganism as described above. The 20 bacterial species belonging to the Gene Ontology (GO) database were used in the analysis. To obtain the list of Q-genes for each microorganism, the frequency of NAU codons of each gene was compared with the average frequency of NAU codons in the whole genome using a right-tail χ^2^ test. FDR correction (α = 0.05) was performed to adjust *P*-values and to avoid identification of false positives (24). Q-genes were submitted to functional enrichment analysis using DAVID and STRING (27,28), and Cluster of Orthologous Genes (COG), GO, KEGG pathways, InterPro, UniProt Keywords, SMART, Pfam and Local STRING network clusters (CL) databases. Terms with a *P*-adjusted value < 0.1 were considered statistically significant.

### Quantification of biofilm formation and cell aggregation in *E. coli*

To quantify biofilm formation and cell adhesion, *E. coli* strains were cultured following a previously reported microtitre plate test with some modifications (29). A 5 ml aliquot of bacterial cultures inoculated with the strains under study was grown overnight at 37°C in LB. Cells were washed twice with M63 medium and the OD_600_ was adjusted to 0.1. For each strain, 10 wells of a sterile 96-well flat-bottom polystyrene hydrophobic plate, without any additional surface treatment (Greiner), were filled with 200 μl of the diluted overnight cultures. The remaining empty wells were filled only with medium. The plates were sealed and incubated at 37°C for 24 h without agitation. The crystal violet staining method was used to quantify biofilm formation and cell adhesion (29). Planktonic cells were removed from bacterial cultures grown in microtitre plates by inverting the 96-well plate and tapping the plate onto a paper towel. To fix adhered bacteria, 200 μl of 99% methanol (Merck) was added to each well, plates were centrifuged at 2500 rpm for 1 min and incubated at room temperature for 20 min. Methanol was removed by inverting the plate and tapping the plate onto a paper towel. The plates were left to dry for 30 min. For biofilm/cell adhesion quantification, wells were filled with 200 μl of 0.1% crystal violet (Sigma-Aldrich), and plates were allowed to stand for 15 min. Wells were washed three times with cold phosphate-buffered saline (PBS) to eliminate the excess dye and plates were air-dried for 15 min. Crystal violet bound to the adherent cells was resolubilized with 200 μl/well of 33% (v/v) glacial acetic acid (PanReac AppliChem ITW Reagents). The absorbance of the obtained solution was measured at 595 nm (OD_595_) using the microtitre plate reader SPECTROstar Nano (BMG Labtech), and OD_595_ values were further normalized against the control condition. To analyse cell aggregation, bacterial cultures were grown in microtitre plates in M63 liquid medium at 37°C for 24 h without agitation. Aggregation was quantified by calculating the percentage of the surface area of each well that was occupied by bacterial aggregates (aggregation area) using ImageJ software (30).

### Extraction and quantification of lipopolysaccharides

Lipopolysaccharides (LPSs) from *E. coli* ST131 pSKII+ and ST131 *queF* were extracted following the Hitchcock and Brown preparation method (31). A 5 ml aliquot of liquid cultures of LB-AMP was grown at 37°C overnight. Cells were washed twice with M63-AMP liquid medium, diluted to an OD_600_ of 0.02 and incubated for 24 h at 37°C. After centrifugation of the cell cultures at 16 000 *g* for 1 min, cell pellets were washed four times with PBS and cell suspensions were diluted with PBS to adjust the OD_600_ to 0.45. Cells from 1 ml of adjusted cultures were sedimented by centrifugation (16 000 *g*, 1 min), the pellets were resuspended in 0.25 ml of lysis buffer [1 M Tris pH 6.8, 10% (v/v) glycerol, 2% (w/v) SDS] and cells were incubated at 100°C for 20 min. Then 30 μg of proteinase K were added followed by an incubation at 25°C for 16 h. For LPS quantification, 10 μl of each sample was mixed with gel loading buffer and loaded onto a 0.75 mm thick, 12% acrylamide SDS–polyacrylamide gel elctrophoresis (PAGE) gel, which was run at 150 V for 1 h in standard Tris–glycine SDS running buffer. LPS bands were visualized by silver staining with a Plus One™ Silver Staining kit (GE Healthcare), and the sum of intensities of all LPS bands for each sample was calculated using ImageJ software (30).

### Sporulation efficiency assay in *B. subtilis*

Pre-cultures of the *B. subtilis* strains PY79 and PY79 Δ*queF* were grown until exponential phase (OD_600_ = 1) in MSgg liquid medium at 37°C and shaking at 200 rpm. They were used to inoculate three 20 ml cultures of each strain in the same medium (initial OD_600_ of 0.05), which were incubated at 37°C and 200 rpm for 5 days. At different time points, 2 ml samples were divided into two fractions: one was incubated at 80°C, to kill vegetative cells but not spores, and the other was incubated at 25°C for 20 min. The number of spores per ml and total colony-forming units per ml (CFU/ml) were calculated from each sample. Percentage sporulation was calculated by dividing the number of spores by total CFUs.

### Biofilm formation analysis in *B*.*subtilis*

*B. subtilis* strain NCIB 3610 and mutant derivatives Δ*queF* and Δ*queF amyE*:*queF* were grown to late exponential phase (OD_600_ = 2) in MSgg liquid medium at 30°C. Drops of 3 μl were spotted on MSgg-agar in the absence or presence of 100 nM preQ_1_ (Sigma-Aldrich). Plates were incubated for 16 h at 30°C. Colony diameter was measured using ImageJ software (30).

### Interbacterial competition assay

*In vitro* competition assay was performed on M63-KAN plates according to the previously reported protocol (32). *P. putida* KT2440 harbouring pSEVA2313 and pSEVA2313/*queF* (predators), and *E. coli* DH10B pSEVA2313 (prey) were grown overnight in LB-KAN medium at 30°C. Cells were washed twice with M63-KAN liquid medium, diluted to an OD_600_ of 0.02 and incubated overnight at 30°C. *E. coli* DH10B was transformed with pSEVA2313 so that it could be co-cultured with *P. putida* in the presence of KAN. Overnight bacterial cultures were adjusted to OD_600_ of 1.0 and mixed in a 1:1 ratio. A 20 μl aliquot of each mixed culture was spotted in triplicate on M63-KAN agar plates and incubated at 30°C for 5 h. Cells were collected, and 10-fold serial dilutions of the different assays were plated onto LB and LB-STR plates to quantify total CFUs and *E. coli* CFUs, respectively. *P. putida* CFUs were calculated by subtracting *E. coli* CFUs from the total CFUs. Relative fitness (F) was calculated as the ratio of the effective growth rates or Malthusian parameters (m) of the two competing strains as determined by Equations (1) and (2):


(1) \begin{equation*}m = lo{g_2}\left( {{\raise0.7ex\hbox{${CF{U_f}}$} \!\mathord{\left/ {\vphantom {{CF{U_f}} {CF{U_i}}}}\right.} \!\lower0.7ex\hbox{${CF{U_i}}$}}} \right)\end{equation*}

(2) \begin{equation*}F = {\raise0.7ex\hbox{${{m_{P.\ putida}}}$} \!\mathord{\left/ {\vphantom {{{m_{P.\ putida}}} {{m_{E.\ coli}}}}}\right.} \!\lower0.7ex\hbox{${{m_{E.\ coli}}}$}}\end{equation*}

where CFU_i_ and CFU_f_ are initial and final cell densities (33). To eliminate the possibility that the effects on interbacterial competition could be due to differences in the growth of *P. putida* overexpressing *queF*, overnight cultures in M63-KAN of *P. putida* pSEVA2313 and pSEVA2313/*queF* were adjusted to OD_600_ of 0.1 in 20 ml and cultured at 30°C until stationary phase.

### Bioinformatic analysis of Q biosynthetic genes

Q biosynthetic genes were evaluated in bacteria included in the COG database (34) in January of 2022. COG annotations corresponding to each Q biosynthetic gene were used to predict the presence of these genes in the genomes of 1134 bacterial species: *queD* (COG0720), *queE* (COG0602), *queC* (COG0603), *queF* (COG0780 and COG2904), *tgt* (COG0343), *queA* (COG0809) and *queG*/*queH* (COG1600 and COG1636). Microorganisms were predicted to synthesize Q *de novo* or salvage Q precursors depending on the absence of *tgt* (non-Q); the presence of *queC*, *queF*, *tgt*, *queA* and *queG*/*queH* (Q *de novo*); the presence of *tgt* and the absence of *queA* and *queG*/*queH* (q salvage); the presence of *tgt*, *queA* and *queG*/*queH* and the absence of *queF* (preQ_1_ salvage); and the presence of *queF*, *tgt*, *queA* and *queG*/*queH* and the absence of *queC* (preQ_0_ salvage) (6). Percentages of non-Q, Q-sources and Q-sinks per phylum were calculated. For NAU codon usage analysis, datasets were obtained by calculating the average frequency of NAU codons in the whole genome of bacteria that may use Q listed in Supplementary Data S2 and S3, or non-Q bacteria listed in Supplementary Data S4A that were contained in the Kazusa database (25).

### Meta-analysis of human gut microbiota metagenomic studies

Metagenomic studies of human gut microbiota of inflammatory bowel disease (IBD) or colorectal cancer (CRC) patients that provided data of the relative abundances of all detected species were considered for the meta-analysis (35–49). BioProyect accession numbers of these studies are available in Supplementary Data S5. Detected species were classified per phylum. Variations in relative abundance between patients and healthy controls were calculated for each species. The sum of differences in relative abundance of all the species of each phylum was calculated. Considering the Q-source and Q-sink proportions (Supplementary Data S4B) and the differences in relative abundance per phylum, total variations in relative abundance of Q-sources and Q-sinks could be estimated (Supplementary Data S5).

### Statistical methods

A right-tail χ^2^ test followed by FDR correction was performed to identify Q-genes from different organisms. Statistical χ^2^ was calculated using Equation (3):


(3) \begin{eqnarray*}{\chi ^2} = \frac{{{{\left( {NA{U_O} - NA{U_E}} \right)}^2}}}{{NA{U_E}}} + \frac{{{{\left( {NonNA{U_O} - NonNA{U_O}} \right)}^2}}}{{NonNA{U_E}}}\nonumber\\ \end{eqnarray*}

where NAU_O_ is the observed number of NAU codons, NAU_E_ is the expected number of NAU codons, calculated by multiplying the number of total codons by the genome-wide frequency of NAU codons, Non-NAU_O_ is the observed number of total codons except NAU codons, and Non-NAU_E_ is the expected number of total codons except NAU codons, calculated by multiplying the number of total codons by the genome-wide frequency of total codons except NAU codons. Statistical χ^2^ was submitted to right-tail χ^2^ distribution for obtaining *P*-values. FDR correction (α = 0.05) was performed to adjust *P*-values and avoid the identification of false positives (24). GraphPad Prism version 7.00 (GraphPad Software, La Jolla, CA, USA) was used to calculate statistical significance and other parameters, including the values of mean and standard deviation (SD) based on the datasets from independent experiments. Statistical tests and parameters are indicated in the figure legends. Statistical significance was calculated using one-way analysis of variance (ANOVA) for multiple comparisons, and one- or two-sided unpaired Student's *t*-test for single variable comparisons.

## RESULTS AND DISCUSSION

### tRNA Q-modification affects the translational efficiency of Q-genes *in vivo* in bacteria

**Figure 2.**
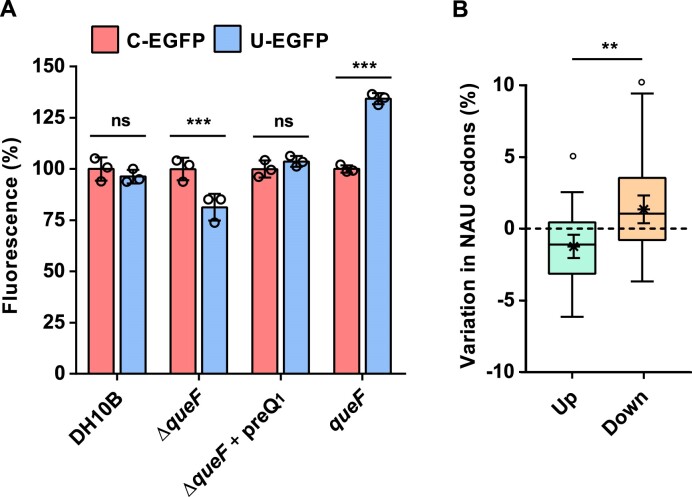
Q affects translation of Q-genes in bacteria. (**A**) Fluorescence intensity of the DH10B, DH10B Δ*queF* (Δ*queF*) and DH10B pSKII+/*queF* (*queF*) strains expressing the gene encoding EGFP in which all NAC/U codons were replaced either by NAC (C-EGFP) or by NAU (U-EGFP). Cultures were incubated at 37°C for 24 h in M63 medium. DH10B Δ*queF* was cultured in the presence or absence of 100 nM preQ_1_. BugBuster® reagent (Novagen) was used to prepare protein extracts from 1 ml of each culture. Fluorescence measurements were adjusted with OD_600_ values and further normalized against the clone of each strain that harboured the C-EGFP construct. Data represent the mean ± SD of three independent experiments performed in triplicate. Differences between U-EGFP and C-EGFP clones of each strain were analysed by two-sided, one-way ANOVA with Sidak's test (***P< 0.001). (**B**) Difference between the genome-wide frequency of NAU codons in *E. coli* (7.7%) and average frequency of NAU codons of genes that encoded up- and down-regulated proteins in the DH10B Δ*queF* mutant relative to the DH10B strain. Boxes represent the median ± interquartile range (IQR), and whiskers denote observations within ± 1.5 times the IQR. Differences between average frequencies of NAU codons were analysed by two-sample *t*-test (**P< 0.01). Asterisks and their error bars represent the mean ± 95 confidence interval, which were used to reveal the displayed significant differences against the average frequency of NAU codons in the whole genome of *E. coli* (°*P*< 0.05).

It has been reported that the presence of Q in tRNAs may enhance translational speed at NAU codons and prevent the codon bias shown by unmodified tRNAs harbouring a GUN anticodon. Therefore, the translational efficiency of Q-genes could be especially affected *in vivo* by altering Q availability. Recently published data point in that direction in the eukaryote *T. brucei* and in human cells (16,17). However, there is still no experimental evidence that Q can regulate Q-gene translation in bacteria. Therefore, we chose *E. coli* as the model bacterial organism to experimentally address that hypothesis. Briefly, two variants of the gene encoding EGFP were synthesized, in which the 51 NAC/U codons were all replaced by either NAC (C-EGFP) or NAU (U-EGFP; Q-gene). These codon-modified versions of the EGFP gene were expressed in three *E. coli* DH10B strains: the wild type, which can synthesize Q, a Δ*queF* mutant, which lacks Q-modified tRNAs (6,50), and pSKII+/*queF*, which overexpresses *queF* and may increase Q biosynthesis (Figure 1). Data revealed that the Δ*queF* mutant exhibited 20% less fluorescence when expressing U-EGFP compared with C-EGFP, while there was no difference when expressed in the wild type (Figure 2A). This phenotype was reversed when the Δ*queF* mutant strains were cultured in the presence of 100 nM preQ_1_ (Figure 2A). Furthermore, the strain overexpressing *queF* (pSKII+/*queF*) showed 34% more fluorescence when harbouring the U-EGFP gene compared with C-EGFP (Figure 2A). No differences in fluorescence were observed between the wild type, Δ*queF* and Δ*queF* + preQ_1_ strains expressing C-EGFP (Supplementary Figure S1), which would rule out indirect effects of the *queF* mutation on plasmid copy number, or on the cell lysis efficiency. Overall, these results suggest that the translational efficiency of NAU codons is reduced in the absence of Q and enhanced when Q biosynthesis is increased. To further support this hypothesis, a comparative proteomic analysis of the DH10B wild-type and Δ*queF* strains was performed. We observed that the average frequency of NAU codons was higher in genes coding for down-regulated proteins than in those coding for up-regulated proteins in the Δ*queF* mutant and that of all *E. coli* genes (Figure 2B; Supplementary Data S1). In summary, experimental evidence suggests that the translation of Q-genes could be affected by the levels of Q in bacteria and highlights the role of Q and possibly other tRNA post-transcriptional modifications in the control of gene expression.

### Biofilm formation and virulence are predicted to be controlled by Q in bacteria

**Figure 3.**
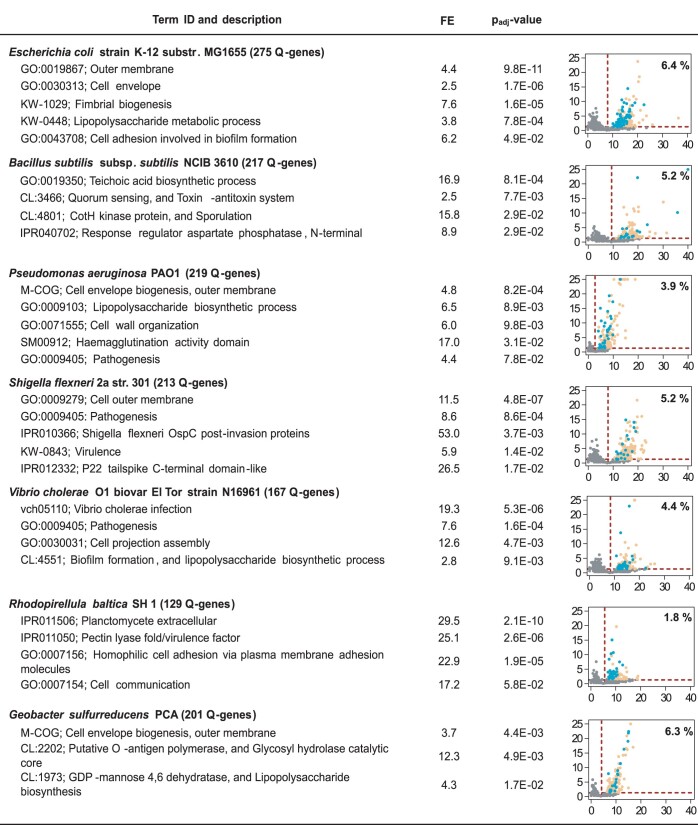
Q is predicted to affect adhesion, biofilm formation and/or virulence in bacteria. Bioinformatic analysis was used to predict Q-genes for each microorganism, which were then submitted to functional enrichment analysis. The number of Q-genes is shown together with the name of each microorganism. Term ID and description of significant ontology terms related to adhesion, biofilm formation and/or virulence are shown, followed by their fold enrichments (FEs) and adjusted *P*-values (p-adj values). The most significant ontology terms related to these processes of seven bacterial species are shown. The results obtained from the other bacterial species and the complete lists of ontology terms are detailed in Supplementary Data S3. Dot plots show the NAU codon frequency of each gene (%; *x*-axis) and their –log_10_ p-adj value corresponding to the right-tail χ^2^ test (FDR = 0.05) used to identify the Q-genes (*y*-axis). Q-genes belonging to significant ontology terms related to adhesion, biofilm formation and/or virulence are represented as blue dots, the rest of Q-genes as yellow dots and non-Q-genes as grey dots. Red lines represent the thresholds used to identify Q-genes (horizontal line, –log_10_ of p-adj value = 0.05; vertical line, average frequency of NAU codons in the whole genome of each microorganism). The percentage of Q-genes compared with the total number of genes for each microorganism is shown in each dot plot.

To investigate which cellular processes could be affected by Q-tRNA, we first performed a bioinformatic analysis to identify Q-genes in *E. coli* and another 20 bacterial species belonging to different phyla (Supplementary Data S2), by statistical comparison of the frequency of NAU codons of each gene with the average frequency in the corresponding genome (see 'Statistical methods'). The percentage of predicted Q-genes in the genome of each species ranged from 0.7% to 11.3% (average 5%) (Figure 3; Supplementary Data S2). We hypothesize that Q would mainly alter the expression of Q-genes, although we do not discard that Q could also affect the expression of genes with several NAU codons but which are non-statistically enriched in them. Q-genes of each microorganism were submitted to functional enrichment analysis using DAVID and STRING bioinformatics tools (27,28). Ontology terms related to cell adhesion, biofilm formation and/or virulence were found to be enriched in Q-genes for all analysed microorganisms (Figure 3; Supplementary Data S3). We hypothesize that small changes in the translation of several functionally related Q-genes could result in higher alterations of certain cellular processes. Therefore, Q-related processes will depend on the main roles of the Q-genes in each organism. These results support the idea that Q-tRNA could precisely modulate the translation of Q-genes to control biofilm formation and/or virulence across the different bacterial phyla. Considering that the known processes involved in biofilm formation are very different between Gram-negative and Gram-positive bacteria, the identification of a possible general mechanism that affects biofilm formation in all bacteria is of particular relevance for the development of treatments to prevent bacterial infections and the negative impact of biofilm formation on surfaces (51). Furthermore, this translational Q regulation could represent a new mechanism for the coordinated control of the expression of functionally related genes. It is noteworthy that a high frequency of NAU codons has been conserved in genes related to these processes in phylogenetically different bacteria, underlining the transcendence and universality of this additional layer of translational regulation performed by Q-tRNA.

### Changes in Q availability affect biofilm formation, virulence and stress resistance in *E. coli*

**Figure 4.**
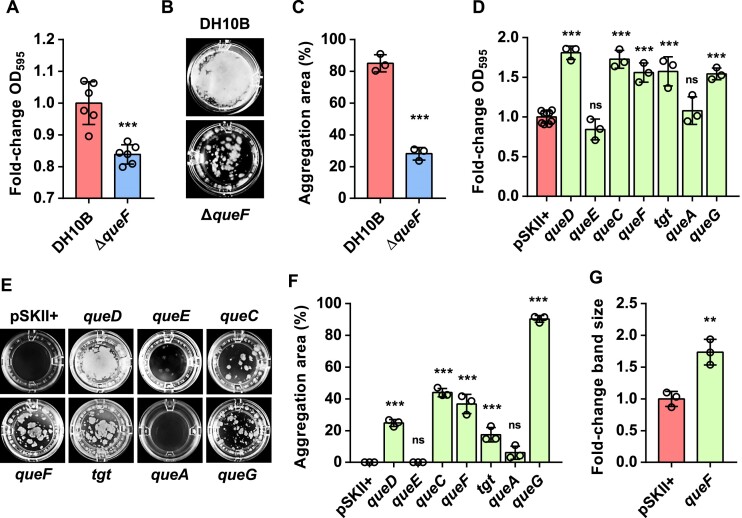
Q affects biofilm formation and virulence in *E. coli*. (**A**–**C**) Biofilm formation (**A**) and cell aggregation (**B** and **C**) are decreased in the absence of Q (Δ*queF*). (**D**–**F**) Overexpression of Q biosynthetic genes from *E. coli* enhanced biofilm formation (**D**) and cell aggregation (**E** and **F**). Cells were cultured overnight in 96-well flat-bottom polystyrene plates at 37°C without agitation in M63 medium supplemented with AMP when required. Biofilm formation was measured following the crystal violet method (29). OD_595_ values were normalized against controls (fold change). Cell aggregation quantification (**C**, **F**) was performed by calculating the percentage of well surface area occupied by cell aggregates (aggregation area). It should be noted that differences in aggregate formation were observed between the controls DH10B strain and DH10B with the empty plasmid (**B**, **E**). These differences may be caused by the toxic effects of the incubation in the presence of an antibiotic, leading to a non-optimal growth and preventing aggregate formation. Data represent the mean ± SD of at least three independent experiments with 10 replicates. Biofilm formation and cell aggregation differences were analysed by two-sample *t*-tests in the case of the Δ*queF* mutant (***P< 0.001) (**A**, **C**), and by a two-sided, one-way ANOVA with Dunnet's test for strains overexpressing Q biosynthetic genes (***P< 0.001) (**D**, **F**). (**G**) LPS production is increased by enhancing Q biosynthesis. *E. coli* ST131 harbouring empty pSKII+ plasmid and ST131 pSKII+/*queF* (*queF*) were grown in liquid M63–AMP medium at 37°C overnight, and LPSs were extracted following the Hitchcock and Brown preparation method (31). LPSs were measured by SDS–PAGE followed by silver staining and band quantification, and LPS measures were normalized against the control (fold change). Data represent the mean ± SD from three independent experiments performed in triplicate. Differences in LPS values were analysed by two-sample *t*-test (**P< 0.01).

To experimentally verify the Q-related processes predicted by the bioinformatics analysis, we first addressed the study of this widely spread translational Q regulation mechanism in a model microorganism, the Gram-negative *E. coli*. Functional enrichment analysis showed that *E. coli* Q-genes were particularly associated with ontology terms related to biofilm formation and virulence, such as ‘Fimbrial biogenesis’, ‘Cell adhesion involved in biofilm formation’ and ‘LPS biosynthetic process’ (Figure 3; Supplementary Data S3) (52). To investigate the effect of Q in these processes, we first tested the ability of an *E. coli* DH10B Δ*queF* mutant to form biofilms and cell aggregates. The *E. coli* K-12 strain (such as DH10B) forms floating cell aggregates at the air–liquid interface when cultured in M63 medium in static or low agitation to promote cell–surface and cell–cell interactions (53). As expected, a reduction in adhesion to the well surface (Figure 4A), and in the formation of cell aggregates (Figure 4B, C), was observed in the Δ*queF* mutant compared with the control DH10B strain. In addition, and consistent with these results, different *E. coli* DH10B clones overexpressing their own Q biosynthetic genes showed more cell adhesion and aggregation compared with the control strain (Figure 4D–F). Moreover, to study the effect of Q on LPS production, the pathogenic *E. coli* ST131 strain was used instead of the non-pathogenic DH10B, which does not produce LPS due to a mutation in the *wbbL* gene (54). We observed that the overexpression of the *queF* gene in ST131 cells produced an increase in the synthesis of LPS compared with those harbouring an empty plasmid (Figure 4G; Supplementary Figure S2A). Altogether, we propose that Q would control biofilm formation and virulence in *E. coli* by affecting the expression of proteins involved in LPS synthesis, adhesion and cell aggregation, as predicted by the bioinformatic analysis. Although we used a static model for evaluation of the effects of Q in *E. coli*, we suggest that Q would also play a similar role in flow systems such as catheters or blood vessels, where pathogenic strains such as ST131 form biofilms. In addition, proteomics results of the DH10B Δ*queF* mutant revealed a decreased expression of proteins RfbC, Glf and flagellin FliC (Supplementary Data S1), which are encoded by Q-genes and involved in LPS biosynthesis and adhesion to both abiotic and biotic surfaces (55). Therefore, the translation of these proteins would be specially affected by Q-tRNA and, therefore, they could be primarily responsible for the observed effects of Q in biofilm formation and virulence in *E. coli*.

It is well known that biofilms represent a protected mode of growth that allows bacteria to survive under hostile conditions (56). Previous studies from our laboratory showed the increase in resistance to different types of stress such as low acidic pH, perchlorate or arsenic in clones of *E. coli* overexpressing Q biosynthetic genes, such as *queF* from *E. coli* and *B. subtilis* or others isolated from environmental microorganisms using functional metagenomics, and indeed we also observed an induction of biofilm formation in all these clones (Supplementary Figure S2B–D) (11,12). Furthermore, comparative proteomics of the DH10B Δ*queF* mutant versus the wild-type strain showed a decreased expression of the transcriptional regulator GadE, encoded by the *gadE* Q-gene, together with several GadE-regulated proteins, which play an important role in the acid stress response (Supplementary Data S1) (57). In conclusion, Q could also be affecting stress responses through the modulation of the expression of genes related to biofilm formation and stress regulators. It cannot be excluded that changes in the expression of certain genes in a Q-lacking *E. coli* strain could be due to additional indirect effects on their transcription (58), since some of the Q-genes encode transcriptional regulators, as in the case of *gadE*.

### Absence of Q impairs sporulation and biofilm formation in *B. subtilis*

**Figure 5.**
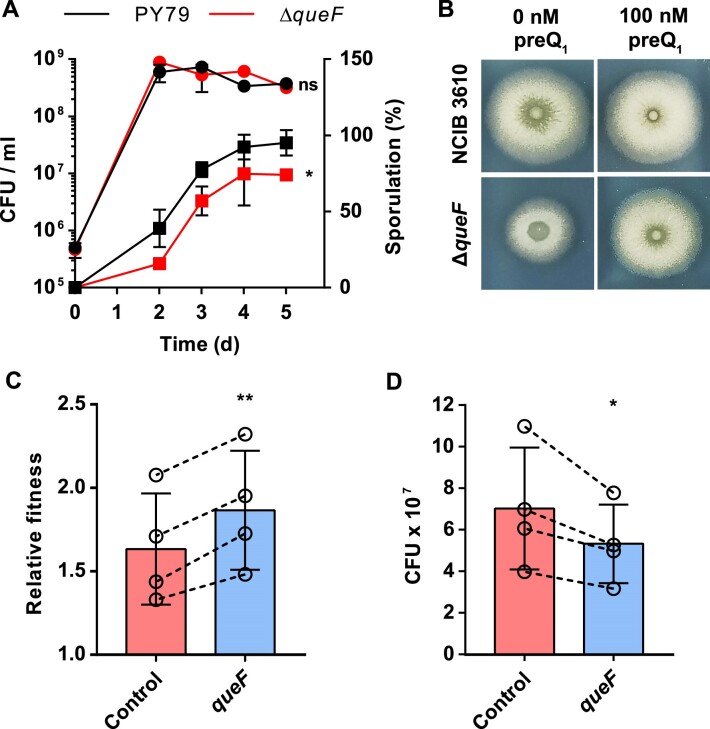
Q affects sporulation and biofilm formation in *B. subtilis*, and interbacterial competition in *P. putida*. (**A**) Absence of Q reduces sporulation efficiency in *B. subtilis*, while cell viability is not affected. PY79 (black) and PY79 Δ*queF* mutant (Δ*queF*; red) strains were cultured in 20 ml of MSgg medium for 5 days at 37°C with agitation. At different time points, cell viability of both strains was determined (circles), 2 ml aliquots of cultures were incubated at 80°C for 15 min to kill vegetative cells and the number of spores was obtained by plate counting. Percentage of sporulation was calculated as the number of spores per total CFUs (squares). Data represent the mean ± SD from three independent experiments with three replicates. Differences between strains were analysed by two-sided, one-way ANOVA (ns: not significant; ***P< 0.001). (**B**) Biofilm formation depends on Q in *B. subtilis*. NCIB 3610 and NCIB 3610 Δ*queF* mutant (Δ*queF*) strains were grown on MSgg-agar medium supplemented or not with preQ_1_ (100 nM) for 16 h at 30°C, and colony sizes were analysed. (**C**) Relative fitness between *P. putida* KT2440 overexpressing its *queF* gene (*queF*) or harbouring the empty pSEVA2313 plasmid (control) used for overexpression (predators), and *E. coli* DH10B harbouring empty pSEVA2313 (prey). *P. putida* and *E. coli* strains were mixed in a 1:1 OD_600_ ratio and cultured on M63-KAN agar for 5 h at 30°C. After incubation, final *P. putida* and *E. coli* CFUs were measured by plate counting, and relative fitness values were calculated (33). (**D**) Number of CFUs of *E. coli* DH10B pSEVA2313 after co-culture incubation with *P. putida* strains. Data represent the mean ± SD of four independent experiments performed in triplicate. Dashed lines indicate paired experimental values. Differences between control and *queF* strains were analysed by paired sample *t*-tests (*P< 0.05; **P< 0.01).

Since the predicted Q-related processes were experimentally verified in *E. coli*, we attempted to achieve the same goal in a completely different model bacterium, the Gram-positive *B. subtilis*. Functional enrichment analysis in *B. subtilis* showed that Q-genes were particularly related to sporulation ontology terms such as ‘CotH kinase proteins’ involved in spore coat formation, and ‘Response regulator aspartate phosphatases’, which control the phosphorelay for sporulation initiation (Figure 3; Supplementary Data S3) (59,60). In fact, we verified that the Δ*queF* mutation in *B. subtilis* strain PY79 decreased sporulation efficiency by 26% compared with the wild-type strain, whereas cell viability remained comparable (Figure 5A). Furthermore, genes related to teichoic acid biosynthesis were also found to be enriched in NAU codons (Figure 3; Supplementary Data S3). Since it has been reported that cells deficient in teichoic acids have a reduced capacity for biofilm formation (61), we analysed the effect of the Δ*queF* mutant on biofilm formation in the undomesticated strain *B. subtilis* NCIB 3610. Unlike the laboratory strain PY79, this strain produces robust and complex biofilms with aerial structures called fruiting bodies (62). We observed that the *B. subtilis* NCIB 3610 Δ*queF* mutant formed smaller colonies than the wild-type strain, as well as other previously described mutants that affect biofilm formation (62), without affecting its growth in liquid medium (Figure 5B; Supplementary Figure S3A, B). As expected, addition of preQ_1_ to the medium or reintroduction of *queF* reversed that phenotype (Figure 5B; Supplementary Figure S3B, C). Altogether, we have shown that changes in Q availability affect biofilm formation and sporulation in *B. subtilis*, and we hypothesize that this phenomenon may be due to the Q-dependent translational regulation of Q-genes involved in those processes, as supported by bioinformatic analysis.

### Q affects the interaction of *P. putida* and *S. meliloti* with other organisms

We attempted to explore whether Q-related processes predicted by bioinformatics in other bacteria could be experimentally verified. Functional enrichment analysis performed in *P. putida* KT2440 strain uncovered that Q-genes were especially related to the ‘Rhs repeat-associated core’ term (Supplementary Data S3). Rhs proteins and Rhs repeat-associated core domain proteins are polymorphic toxins involved in contact-dependent growth inhibition (63). These proteins are secreted through a type VI secretion system (T6SS), a macromolecular machine that delivers toxic proteins to neighbouring cells (64). Competition assays were performed to test whether availability of Q regulated the capacity of *P. putida* to inhibit the growth of other bacteria. For this purpose, *E. coli* DH10B cells were co-cultured as prey with *P. putida* KT2440 cells overexpressing *queF* as predators, and the relative fitness of *P. putida* over *E. coli* was calculated. We observed that the growth inhibition of *E. coli* by *P. putida* increased when *queF* was overexpressed in the latter, which correlates with the decrease in the *E. coli* cell number after co-culture incubation (Figure 5C, D). It should be noted that *queF* overexpression in *P. putida* did not affect its growth or cell viability after co-cultivation (Supplementary Figure S3D, E). Therefore, the interbacterial competition capacity of *P. putida* may be increased by the effect of Q, through the enhanced translation of genes encoding Rhs repeat-associated core proteins.

Another bacterium in which the availability of Q affects its interaction with other organisms is the model rhizobium *S. meliloti*. Marchetti *et al.* experimentally showed that when the legume *Medicago truncatula* was inoculated with *queF* mutants of *S. meliloti*, nitrogen fixation was profoundly impaired, and the random bacteroid organization within nodules indicated a defect in symbiosome organization (10). Our bioinformatic analysis revealed that *S. meliloti* Q-genes were particularly associated with the ontology terms ‘Plasmid’, ‘Haemolysin-type calcium-binding repeat’ and ‘Serralysin-like metalloprotease C-terminal’ (Supplementary Data S3). In rhizobia, genes involved in establishing symbiosis, nodulation and nitrogen fixation (*nod*, *nif*, and *fix*) are known to be usually located in one or more plasmids called pSyms (65), and 22 out of 36 *S. meliloti* Q-genes were harboured in these large plasmids. Furthermore, haemolysin-type calcium-binding proteins of rhizobia have been previously shown to be related to Nod proteins, which are essential for legume nodulation and host specificity, and serralysins might be necessary for survival in their plant hosts (66,67). For these reasons, and considering that *nifK* is one of the Q-genes of *S. meliloti* and codes for a nitrogenase subunit (Supplementary Data S2), we propose that *S. meliloti queF* mutants may exhibit impaired nodule cell infection and nitrogen fixation due to a decrease in the translation of essential factors involved in these processes. In summary, as predicted by bioinformatics, virulence and interbacterial competition in *P. putida*, symbiosis and nodule infection in *S. meliloti* and, in general, the interaction of bacteria with other organisms could be affected by Q through changes in the expression of Q-genes involved in those processes.

### Q is involved in the expression of virulence factors in human pathogenic bacteria

The bioinformatics and experimental results described above allow us to propose that Q affects biofilms and virulence widely in bacteria, and we have shown that the presence of Q enhances biofilm formation in Gram-negative and Gram-positive bacteria, and particularly induces the expression of virulence factors (LPSs) in the pathogenic *E. coli* ST131 strain and toxins involved in interbacterial competition in *P. putida*. Therefore, we hypothesize that Q could be playing similar roles generally in human pathogens. In fact, ontology terms directly related to pathogenesis and/or virulence were significantly enriched in Q-genes throughout all human pathogens analysed (Figure 3; Supplementary Data S3): (i) ‘Pathogenesis’ in *S. flexneri*, *Pseudomonas aeruginosa*, *Salmonella enterica* and *Vibrio cholerae*; (ii) ‘Infection’ in *Staphylococcus aureus* and *V. cholerae*; and (iii) ‘Virulence’ in *S. flexneri*, *S. enterica*, *V. cholerae* and *Yersinia pestis*. In *Streptococcus pneumoniae*, an enrichment was observed in Q-genes coding for ‘Cell wall/choline-binding repeat’ proteins, including *lytB, lytC, cbpD, cbpE* and *cbpG* (Supplementary Data S3). These pneumococcal proteins are involved in the release of toxic compounds that damage the host tissues, in cellular adhesion and in colonization, and mutants in the genes that encode them have decreased virulence and colonization of the nasopharynx (68). In addition, in *Listeria monocytogenes*, several terms related to virulence, adhesion, invasion and pathogenesis were enriched in Q-genes, especially ‘Internalin, N-terminal domain’ and ‘Gram-positive LPxTG cell wall anchor protein’ (Supplementary Data S3). Internalins are bacterial surface proteins necessary for the internalization of *L. monocytogenes* within intestinal epithelial host cells (69), and LPxTG cell wall anchor proteins help to perform the first attachment to the host tissue, enabling *L. monocytogenes* and other Gram-positive pathogens to mount successful infections (70). Finally, listeriolysin O, encoded by the Q-gene *hly*, is required for intracellular multiplication and infection dissemination (71). In *Neisseria meningitidis*, bioinformatic analysis showed enrichment in the ‘Pectin lyase fold/virulence factor’ term, which is related to regulation of adhesion to mammalian cells (72), and in terms such as ‘VENN motif-containing domain’, ‘Haemagglutinin/haemolysin putative’ and ‘Filamentous haemagglutinin’, which are constituted by a repertoire of proteins called polymorphic toxins (Supplementary Data S3) (73). The complex microbiota of the nasopharyngeal mucus limits the colonization and growth of *N. meningitidis*, but polymorphic toxins inhibit the local microbiota in a contact-dependent manner (73). Therefore, Q may increase the translation of this polymorphic toxin which would enhance virulence and interbacterial competition, similarly to what we demonstrated in *P. putida* (Figure 5C, D). Finally, in the case of *Helicobacter pylori*, bioinformatic analysis revealed enrichment in ‘Autotransporter beta-domain’ proteins encoded by the Q-genes *vacA*, *HP_0289* and *HP_0922* (Supplementary Data S3). These proteins mediate adhesion, invasion, intracellular movement, agglutination and biofilm formation (74). Thus, the bioinformatic analysis predicted that changes in Q availability may play an important role in the expression of certain virulence factors and, therefore, the pathogenicity of different types of human pathogens.

It has previously been shown that q affects virulence of *S. flexneri*, an enteropathogen responsible for bacillary dysentery (shigellosis), and *tgt* mutants of *S. flexneri* are unable to invade host cells by markedly reducing the translation of VirF, a key transcriptional regulator of virulence factors required for cellular invasion and spreading of this pathogen (9). In fact, potent TGT inhibitors have been designed as new specific drugs against shigellosis (75). The functional enrichment analysis performed in *S. flexneri* showed that ‘Pathogenesis’ and ‘Virulence’ terms were enriched in Q-genes (Figure 3; Supplementary Data S3). In addition, VirF is encoded by the Q-gene *virF*, which could explain the decrease in *virF* translation observed when *tgt* is mutated (Supplementary Data S2). Furthermore, we found an enrichment in Q-genes coding for *S. flexneri* outer surface protein C (OspC), which may be involved in post-invasion events related to virulence, and for Pectin lyase fold/virulence factors, which mediate adhesion to target mammalian cells (Figure 3; Supplementary Data S3) (72,76). In summary, these data indicate that pathogenesis is affected by Q in *S. flexneri*, and support the idea that this phenomenon generally occurs in human pathogens, as predicted by the bioinformatic analysis. In this sense, inhibitors of bacterial TGT or other bacterial Q biosynthesis enzymes may represent novel drugs to treat not only shigellosis, but also other bacterial infections.

### Imbalance between Q-source and Q-sink bacterial populations may alter the functionality of microbiomes

**Figure 6.**
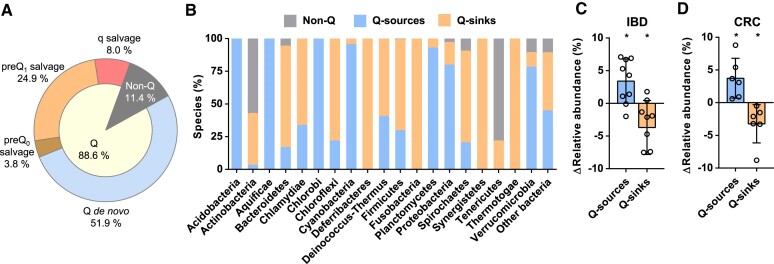
Classification of bacteria depending on whether they use and/or produce Q or not, and the alteration of these populations under microbial dysbiosis. (**A**) Analysis of the presence or absence of Q biosynthetic genes in 1134 bacterial species was analysed using the COG database. These microorganisms were classified depending on: the absence of *tgt* (non-Q), the presence of *queC*, *queF*, *tgt*, *queA* and *queG*/*queH* (Q *de novo*), the presence of *tgt* and the absence of *queA* and *queG*/*queH* (q salvage), the presence of *tgt*, *queA* and *queG*/*queH* and the absence of *queF* (preQ_1_ salvage), the presence of *queF*, *tgt*, *queA* and *queG*/*queH* and the absence of *queC* (preQ_0_ salvage). Microorganisms that were predicted to synthesize Q *de novo* or salvage Q precursors were considered as Q-sources or Q-sinks, respectively. (**B**) Proportion of Q-source (blue), Q-sink (orange) and non-Q (grey) species classified by phylum. Some phyla, such as *Acidobacteria*, *Aquificae*, *Chlamydiae* or *Fusobacteria*, are represented by a low number of species, making it difficult to draw general conclusions (Supplementary Data S4). (**C** and **D**) Human gut microbiota of IBD (**C**) and CRC (**D**) patients shows a decrease in Q-sinks and an increase in Q-sources. Metagenomic studies of human gut microbiota of IBD or CRC patients that provided data of the relative abundances of all detected species were considered for the meta-analysis (35–49). Detected species were classified per phylum. Variations in relative abundance between patients and healthy controls were calculated for each detected species. The sum of differences in relative abundance of all the species of each phylum was calculated. Considering the proportions of Q-sources and Q-sinks (Supplementary Data S4B) and the differences in relative abundance per phylum, total variations in relative abundance of Q-sources and Q-sinks could be estimated (Supplementary Data S5). Data represent variations in the relative abundance of Q-source and Q-sink bacteria together with the mean and SD. Significance of Q-source and Q-sink relative abundance variations was analysed by two-sided, one-sample *t*-test (*P< 0.05; **P< 0.01).

Due to the predicted general role of Q in biofilm formation, virulence, pathogenesis and other specific processes, and given that bacteria can synthesize Q *de novo* or salvage Q precursors, we wondered about the ecological relevance of Q biosynthetic genes in nature. The presence of these genes was analysed in the 1134 bacterial species of all phyla that are included in the COG database (34) (Supplementary Data S4). It was shown that 88.6% of them harbour the *tgt* gene, so they could use Q for tRNA modification (Q-bacteria). *Actinobacteria* and *Tenericutes* phyla contained most of the species that may not use Q (non-Q bacteria) (Figure 6A, B; Supplementary Data S4). In fact, NAU codons usage of non-Q bacteria was observed to be reduced compared with that of Q bacteria, except for *Tenericutes*, animal and plant parasites that might import Q-tRNAs from cell host as mitochondria do (Supplementary Figure S4) (77). Moreover, the 51.9% of analysed species could synthesize Q *de novo* (Q-sources), whereas the 36.7% would salvage Q precursors (Q-sinks). The main Q-sources would be *Proteobacteria* and *Cyanobacteria*, while *Firmicutes*, *Bacteroidetes* and *Actinobacteria* could represent the main Q-sinks (Figure 6A, B; Supplementary Data S4). Only 4% of bacterial species analysed were predicted to salvage preQ_0_, a high proportion (25%) would salvage preQ_1_ and 8% would salvage q (Figure 6A; Supplementary Data S4). Therefore, microbiomes will be composed of Q-source and Q-sink populations maintaining a close interaction. Several pieces of evidence in bacteria and eukaryotes suggest that Q precursors can be salvaged from the environment (Figures 2A and 5B; Supplementary Figure S3B) (5,78,79). Thus, we propose that Q-sinks would salvage Q precursors produced by Q-sources. In this sense, considering the relevance of Q in the control of processes related to inter- and intraspecific cell–cell interactions, we hypothesize that the proper structure and functionality of a microbiome would be conditioned by an adequate balance between Q-source and Q-sink populations. Alterations in this balance, for example caused by the invasion of other microorganisms, would affect the Q availability and thus the dysregulation of Q-related processes in different species of a microbiome.

To explore this hypothesis, we chose a microbial community as complex as the human gut microbiome, and we analysed the changes in Q-source and Q-sink populations under conditions of microbial dysbiosis. IBD is a chronic inflammatory disorder of the intestinal tract of an unknown cause, which includes Crohn's disease and ulcerative colitis. This disease is commonly associated with microbiome dysbiosis, with a decrease in *Faecalibacterium prausnitzii, Roseburia* sp., *Bifidobacterium* sp., Groups IV and XIVA *Clostridium* or *Bacteroides*, and an enrichment in *E. coli*, *Ruminococcus* sp., *Veillonellaceae*, *Pasteurellacaea*, *Enterobacteriaceae* and other *Proteobacteria* (80). We observed that the decreased bacterial species were predominantly Q-sinks, whereas those undergoing enrichment positively correlated with IBD were Q-sources (Supplementary Data S4A). This was supported by a meta-analysis of metagenomic studies evaluating differences in human gut microbiota composition in IBD patients, which showed a significant decrease in the relative abundance of Q-sinks and an increase in Q-sources (Figure 6C; Supplementary Data S5). The same meta-analysis was performed with metagenomic studies that analysed the relevance of the gut microbiome in CRC, another disease closely related to gut microbiome dysbiosis (81), and we obtained similar results to those in IBD (Figure 6D; Supplementary Data S5). Moreover, it is known that the prognosis of IBD or intestinal dysbacteriosis may be improved by supplementing probiotics with *Lactobacillus* sp. or *Bifidobacterium* sp. (82,83), which are Q-sink bacteria (Supplementary Data S4A). Although these are preliminary data and further experimental evidence is needed, we propose that a reduction of Q-sinks and an enrichment of Q-sources derived from microbial dysbiosis could lead to increased bacterial virulence through a higher Q availability, and to the promotion of certain disorders in the gut such as IBD or CRC. This hypothesis would open the door to explore whether controlling Q availability may help to regulate the functionality of the gut microbiome or other microbial communities.

In summary, we conclude that: (i) tRNA Q-modification would be controlling the translation of genes enriched in NAU codons (Q-genes) in bacteria, as we suggest by *gfp* gene recoding and by proteomic experiments of a Δ*queF* mutant in *E. coli*, and as reported in eukaryotes in several previous studies (14–17); (ii) using a bioinformatic approach based on the assumption that Q-tRNAs control Q-genes expression, Q-tRNAs were proposed to be involved in the control of the expression of genes related to cell adhesion, biofilm formation, virulence and, in general, in processes related to cell–cell and cell–surface interactions in most of the bacterial species analysed from various phyla; and (iii) some of the predictions in the three model bacteria *E. coli*, *B. subtilis* and *P. putida* were experimentally validated, supporting that Q-tRNAs control Q-genes expression. Altogether, we hypothesize that Q controls biofilm formation and virulence through orchestrated translational regulation of functionally related genes enriched in NAU codons and involved in those biological processes. Further studies will be required to confirm that the translation of the predicted Q-genes in different bacterial species is directly regulated by Q-tRNAs.

Furthermore, the regulatory mechanisms involved in biofilm formation and virulence vary based on species-specific physiology, which hinders the development of general treatments to control infections and biofilm formation on surfaces of interest. One of these mechanisms is quorum sensing, which is mediated by completely different extracellular signalling molecules and pathways in Gram-negative (acylated homoserine lactones) and Gram-positive (oligopeptides) bacteria (84). Therefore, we propose tRNA Q-modification as the first identified regulatory mechanism involved in the control of biofilm formation and virulence common to all bacteria, regardless of their taxonomic classification. In this sense, this research may open the door to the development of novel treatments based on inhibition of tRNA Q-modification for treatment of infections caused by any bacterial pathogen, and also to prevent biofouling, biocorrosion and biodeterioration of materials caused by biofilms formed by any bacteria. Moreover, considering that tRNA Q-modification enhances virulence and biofilm formation, we suggest that the imbalance of Q-source and Q-sink populations and the alteration of Q availability would affect the functionality of complex microbial communities, such as the gut microbiome. It is worth noting that this putative mechanism of translational regulation by Q-modified tRNAs would not only represent an additional layer of gene expression regulation, but also may allow for the coordinated control of functionally related genes. Finally, since Q seems to play a similar role in eukaryotes controlling the translational efficiency of NAU codons, the bioinformatics approach described here will be used to predict Q-related processes in different eukaryotic species, which could be subsequently validated experimentally. In this sense, given the effect of Q in relevant physiological processes in bacteria and presumably in eukaryotes, which cannot produce Q, we underline the importance of providing a source of Q or Q precursors especially in the study of Q-sink microorganisms, eukaryotic cell lines and germ-free eukaryotic organisms.

## Supplementary Material

gkad667_Supplemental_Files

## Data Availability

All data are available in Supplementary Data files and from the corresponding authors upon request.
