# Supplementary material for: tRNA queuosine modification is involved in biofilm formation and virulence in bacteria
Source: Nucleic Acids Res. 2023 Aug 28;51(18):9821–37. doi: 10.1093/nar/gkad667 (PMC10570037; doi:10.1093/nar/gkad667)
Supplement: gkad667_Supplemental_Files [file gkad667_supplemental_files.zip › Diaz_Rullo_2023_NAR_Supplementary_Material_Revision_2.pdf]

SUPPLEMENTARY MATERIALS OF THE MANUSCRIPT:

tRNA queuosine modification is involved in biofilm formation and virulence in bacteria.

AUTHORS

Jorge Díaz-Rullo[1]* and José Eduardo González-Pastor[1]*

[1] Department of Molecular Evolution. Centro de Astrobiología (CAB), CSIC-INTA. Carretera de Ajalvir km 4, Torrejón de Ardoz (28850). Madrid, Spain.

*To whom correspondence should be addressed. Tel: +34 91 5206434; Fax: +34 91 5201074; Email: gonzalezpje@cab.inta-csic.es. Correspondence may also be addressed to Jorge Díaz-Rullo. Tel: +34 91 5206471; Fax: +34 91 5201074; Email: jdiaz@cab.inta-csic.es.

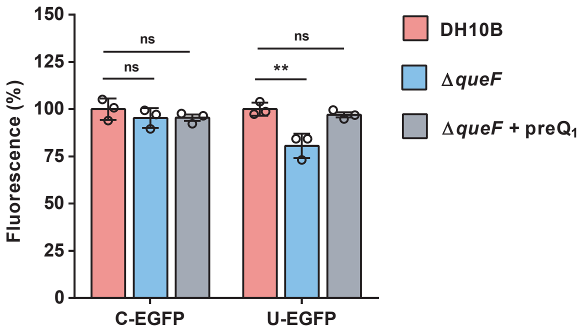

Supplementary Figure S1. Additional data supporting that Q affects translation of Q-genes in bacteria. Fluorescence intensity of the DH10B, DH10B Δ*queF* (Δ*queF*) and DH10B Δ*queF* cultured in the presence of 100 nM $preQ_1$ expressing the gene encoding EGFP in which all NAC/U codons were replaced either by NAC (C-EGFP) or by NAU (U-EGFP). Cultures were incubated at 37 ºC for 24 h in M63. BugBuster® reagent (Novagen) was used to prepare protein extracts from 1 ml of each culture. Fluorescence measurements were adjusted with OD_{600} values and further normalized against the DH10B strain that harboured the C-EGFP or U-EGFP construct. Data represent the mean ± S.D. of three independent experiments performed in triplicate. Differences between strains were analysed by two-sided, one-way ANOVA with Sidak's test (ns: not significant; **p<0.01).

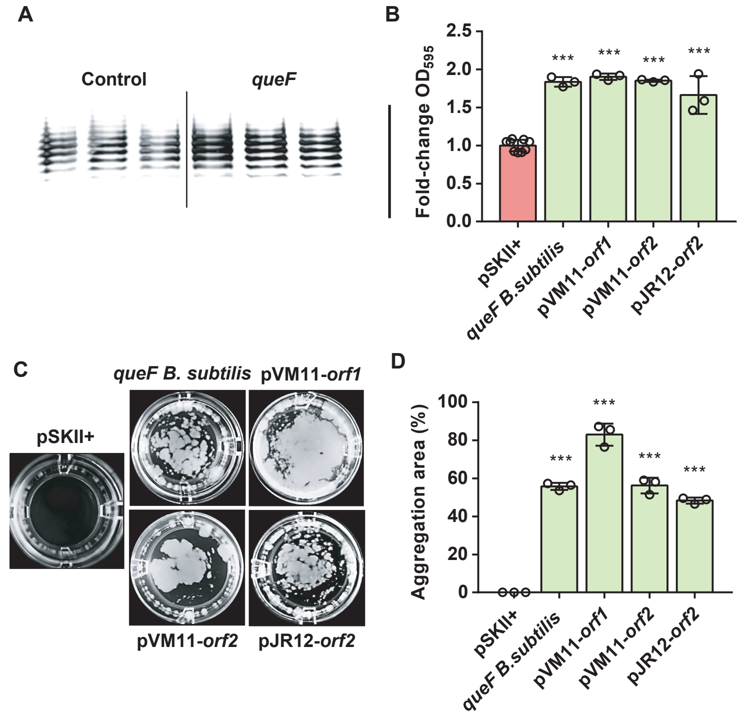

Supplementary Figure S2. Additional data supporting that biofilm formation and cell aggregation are affected by Q in *E. coli*. (**A**) SDS-PAGE followed by silver staining of LPS samples extracted from *E. coli* ST131 harboring empty pSKII+ plasmid and ST131 pSKII+/*queF* (*queF*) grown in M63-AMP liquid medium at 37°C overnight. (**B**-**D**) Biofilm formation (**B**) and cell aggregation (**C**-**D**) are increased in *E. coli* DH10B clones overexpressing Q biosynthetic genes, *queF* from *B. subtilis* and others retrieved in previous studies from environmental microorganisms using functional metagenomics (pVM11-*orf*1, pVM11-*orf*2 and pJR12-*orf*2). Cell aggregation quantification (**D**) was performed by calculating the percentage of well surface area occupied by cell aggregates (aggregation area). OD_{595} values were normalized against controls (fold change). Data represent the mean ± S.D. from three independent experiments with ten replicates. Differences between strains were analyzed by two-sample t-test (***$p<0.001$) (**B**), and two-sided, one-way ANOVA with Dunnet's test (***$p<0.001$) (**D**).

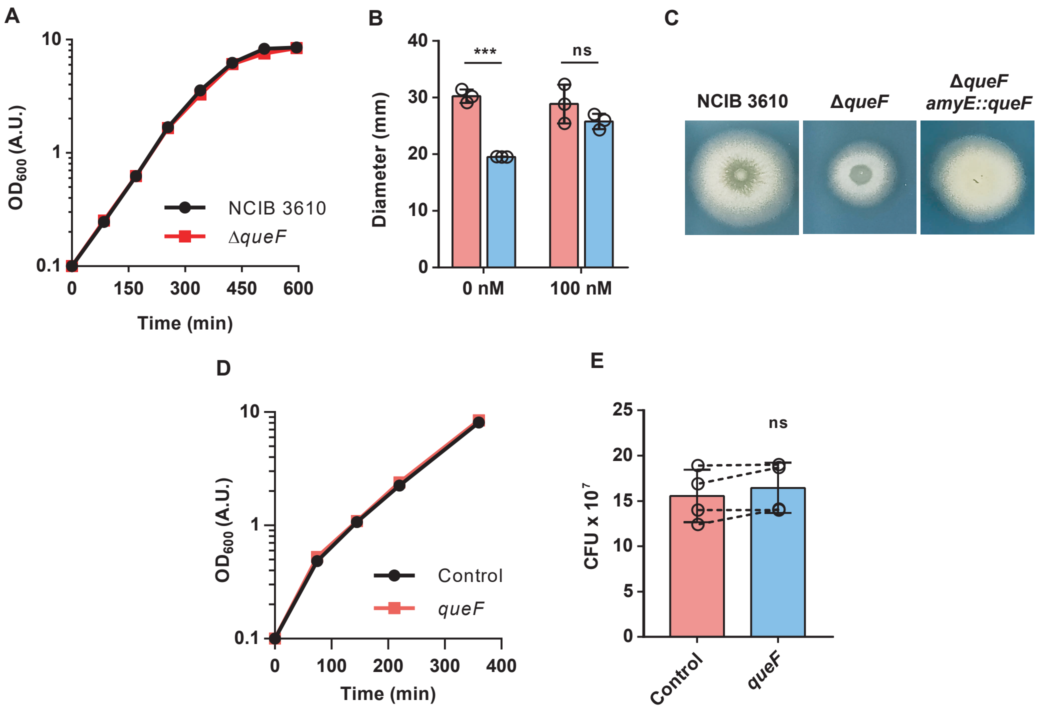

Supplementary Figure S3. Additional data supporting the idea that Q affects sporulation and biofilm formation in *B. subtilis* and interbacterial competition in *P. putida*. (**A**) Growth is not affected when *queF* gene is depleted in *B. subtilis* NCBI 3610 strain. (**B**) Biofilm development depends on Q. *B. subtilis* NCIB 3610 and NCIB 3610 Δ*queF* mutant strains (Δ*queF*) were grown in the absence or presence of 100 nM preQ$_1$. Differences were analyzed by two-sided, two-way ANOVA with Sidak's test (***p<0.001). (**C**) Impairment of biofilm formation capacity of NCIB 3610 Δ*queF* mutant strain is reversed by complementation of *queF* gene. (**D**) Overexpression of the *queF* gene does not affect *P. putida* growth. (**E**) Number of CFUs of *P. putida* KT2440 strains after co-culture incubation with *E. coli* DH10B pSEVA2313. Differences between control and *queF* strains were analyzed by paired sample t-test (ns: not significant). Graphical data represent the mean ± S.D. from three (**A**-**B**, **D**) or four (**E**) independent experiments performed in triplicate.

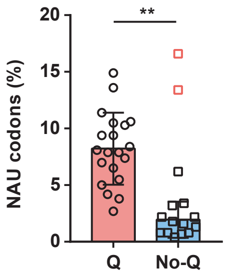

Supplementary Figure S4. Predicted Non-Q bacteria exhibit a reduced NAU codons usage. Data show the average percentage of NAU codons in the whole genomes of bacteria that were predicted to use Q (Q) or not (Non-Q). Red squares represent *Tenericutes*. Data represent the mean ± S.D. NAU codons frequency differences were analyzed by two-sample t-test (**p<0.01).

Supplementary Table S1. Strains used in this work.

Strain	Genotype	Reference
E. coli		
DH10B	F–*mcrA* Δ*(mrr-hsdRMS-mcrBC)* φ80*lacZ*ΔM15 Δ*lacX74 recA1 endA1 araD139* Δ*(ara-leu)7697 galU galK* λ–*rpsL*(Str^R) *nupG*	Invitrogen
Δ*queF*	DH10B with deletion in *queF* (Δ*queF::kan*)	This work
queG	DH10B overexpressing *queG* (pSKII+/*queG*)	This work
queA	DH10B overexpressing *queA* (pSKII+/*queA*)	This work
tgt	DH10B overexpressing *tgt* (pSKII+/*tgt*)	This work
queF	DH10B overexpressing *queF* (pSKII+/*queF*)	Díaz-Rullo et al., 2021
queC	DH10B overexpressing *queC* (pSKII+/*queC*)	This work
queE	DH10B overexpressing *queE* (pSKII+/*queE*)	This work
queD	DH10B overexpressing *queD* (pSKII+/*queD*)	This work
queF B.subtilis	DH10B overexpressing *queF* from *B. subtilis* *(*pSKII+/*queF B.subtilis)*	Díaz-Rullo et al., 2021
pVM11-*orf1*	DH10B overexpressing arsenic resistance gene pVM11-*orf*	Morgante et al., 2015
pVM11-*orf2*	DH10B overexpressing arsenic resistance gene pVM11-*orf2*	Morgante et al., 2015
pJR12-*orf2*	DH10B overexpressing perchlorate resistance gene pJR12-*orf2*	Díaz-Rullo et al., 2021
ST131	Wild type	From R. del Campo laboratory
ST131 *queF*	ST131 overexpressing *queF* (pSKII+/*queF*)	This work
B. subtilis		
NCIB 3610	Wild type	Bacillus Genetic Stock Center
PY79	*swrA srf*	P. Youngman
NCIB 3610 Δ*queF*	NCIB 3610 with deletion in *queF* (Δ*queF::mls)*	This work
PY79 Δ*queF*	PY79 with deletion in *queF* (Δ*queF::mls)*	This work
NCIB 3610 Δ*queF* *amyE*::*queF*	NCIB 3610 with deletion in *queF* (Δ*queF::mls)* and *queF* complementation (*amyE::queF*)	This work
P. putida		
KT2440	Wild type	From V. de Lorenzo laboratory
queF	KT2440 overexpressing *queF* (pSEVA2313/*queF*)	This work

Supplementary Table S2. Primers used in this work. Underlined sequences correspond to restriction sites**.**

Construct	Sequence (5'-3')	Restriction enzyme
E. coli DH10B pSKII+/*queG*	GGTGGTGAGCTCCTTGAATTGCAATGCGTTTTT	SacI
	GGTGGTACTAGTGCGAAGCATCAGCTCATAGA	SphI
E. coli DH10B pSKII+/*queA*	GGTGGTCTCGAGATGCGCGTTACCGATTTCTC	XhoI
	GGTGGTTCTAGACGTCAGAAAAACAGTCCAACG	XbaI
E. coli DH10B pSKII+/*tgt*	GGTGGTCTCGAGCGGTGATGCGATGTTTATCA	XhoI
	GGTGGTTCTAGAAAAAAGCTCATTAAATTTCCCTCA	XbaI
E. coli DH10B pSKII+/*queF*	GGTGGTCTCGAGTAATGCACGGTGAAGGAACA	XhoI
	GGTGGTTCTAGACAGCACGCAAAATTGAGAAA	XbaI
E. coli DH10B pSKII+/*queC*	ACGCGTTCTAGACGACACCCGGAATAATTACCT	XbaI
	GGTGGTCTCGAGGCGGCTGGTTTGAATTTACC	XhoI
E. coli DH10B pSKII+/*queE*	AGCACGTCTAGACTATGCAGTACCCGATTAACGAG	XhoI
	GACAATCTCGAGGTTCCACCCATTTATAACGCTT	XbaI
E. coli DH10B pSKII+/*queD*	GGTGGTCTCGAGATTTTGCGCAACAAAGTCGT	XbaI
	GGTGGTTCTAGAGACCGCCGACACACTCTTAT	XhoI
E. coli DH10B pSKII+/*queF*	GGTGGTCTCGAGTAATGCACGGTGAAGGAACA	XhoI
	GGTGGTTCTAGACAGCACGCAAAATTGAGAAA	XbaI
B. subtilis NCIB 3610 *ΔqueF*	CCGTGCAGCGCTTGAAACTCA	-
	ATTATGTCTTTTGCGCAGTCGGCCTAGCAATGTTACA CCTTCTAATTCTGATTC	-
	CATTCAATTTTGAGGGTTGCCAGCCCATTGACAGAG CTCCTGCAAGATA	-
	CCACTTGTTCCACCCGTTACAAGC	-
B. subtilis NCIB 3610 Δ*queF amyE:queF*	GGTGACGCTAGCCTGATTGCACATTTGCTTGG	NheI
	GAGCATGCATGCCACACTCCGTATCACACAACG	SphI
P. putida KT2440 pSEVA2313/*queF*	GGTGGTGAATTCACTGGCTCTGCAAGGCGGG	EcoRI
	GGTGGTTCTAGAAATAGCGGGGGCCGCAAAGC	XbaI

Supplementary Data S1. Proteomics data comparing DH10B and DH10B Δ*queF* mutant strains. Table shows DEPs Uniprot ID and description, identified peptides, normalized abundances, abundance ratios, p-adj values, frequency of NAU codons of genes encoding for DEPs and whether these genes are Q-genes or not. Green highlighted proteins were encoded by Q-genes. Proteins encoded by genes that are regulated by transcriptional regulator GadE are highlighted with red letters. Differentially expressed proteins (DEPs) were extracted by performing two-sided, two-sample t-test followed by false-discovery rate (FDR) correction (α=0.05). Proteins with an associated p-adjusted value < 0.1 were considered as DEPs.

Supplementary Data S2. Lists of Q-genes of several representative bacteria. Human pathogens are highlighted with red letters. For each microorganism, the percentages of Q-genes over the total number of genes are shown. For each gene, the total number of codons, the number, and the frequency of NAU codons and its p-adj value, and its description are shown.

Supplementary Data S3. Enrichment analysis with Q-genes of several representative bacteria. Human pathogens are highlighted in red. Ontology terms related to adhesion, biofilm formation, virulence and/or other similar processes are highlighted in yellow. When different terms with the same description were enriched, it was selected the term with the lowest p-adj value. When different terms with similar descriptions were enriched, it was selected the most general term that included more Q-genes. When different terms were constituted by the same genes, the term with the highest fold enrichment was selected.

Supplementary Data S4. Classification of bacterial species depending on the presence of Q biosynthetic genes. (A) The presence or absence of Q biosynthetic genes were evaluated in bacteria included in COG database. **(B)** Proportion of Q-sources, Q-sinks and Non-Q bacterial species per phylum.

Supplementary Data S5. Meta-analysis of human gut microbiota metagenomic studies. Variations in relative abundance between patients and healthy controls were calculated for each detected species. The sum of differences in relative abundance of all the species of each phylum was calculated. Considering the Q-sources and Q-sinks proportions (Supplementary Data S4B) and the differences in relative abundance per phylum, total variations in relative abundance of Q-sources and Q-sinks could be estimated.
